# Engaging Stigmatised Communities in Australia with Digital Health Systems: Towards Data Justice in Public Health

**DOI:** 10.1007/s13178-023-00791-6

**Published:** 2023-02-06

**Authors:** Anthony K J Smith, Mark D. M. Davis, James MacGibbon, Timothy R. Broady, Jeanne Ellard, John Rule, Teddy Cook, Elizabeth Duck-Chong, Martin Holt, Christy E. Newman

**Affiliations:** 1 Centre for Social Research in Health (CSRH), UNSW Sydney, Sydney, Australia; 2 School of Social Sciences, Monash University, Melbourne, Australia; 3 Australian Research Centre in Sex, Health and Society (ARCSHS), La Trobe University, Melbourne, Australia; 4 National Association of People With HIV Australia, Newtown, Australia; 5 ACON, Surry Hills, Australia; 6 Kirby Institute, UNSW Sydney, Sydney, Australia; 7 Sydney, Australia; 8 Australian Human Rights Institute, UNSW Sydney, Sydney, Australia

**Keywords:** Consent, Datafication, Data justice, Electronic health records, Privacy

## Abstract

**Introduction:**

In 2018, following government policy changes to Australia’s national electronic health record system, ‘My Health Record’, consumer advocates—including organisations representing people living with HIV, people who use drugs and sex workers—raised concerns about privacy and data security. Responding to these controversies, this study explores the practical, ethical and political complexities of engaging stigmatised communities with digital health systems.

**Methods:**

We conducted 16 qualitative semi-structured interviews in 2020 with key informants representing communities who experience stigma, discrimination and marginalisation in Australia. These communities included people living with HIV, sex workers, people who inject drugs, gay and bisexual men and transgender and gender diverse people. We conducted a reflexive thematic analysis.

**Results:**

Key informants were sceptical of proposed benefits of electronic health records for their communities, and concerned about privacy risks and the potential for discrimination. Meaningful consultation, consent mechanisms and tackling structural stigma were raised as solutions for engaging communities.

**Conclusions:**

Although communities could benefit from being included in digital health systems, significant cultural, legal and social reforms from government were believed to be necessary to build trust in digital health systems. We argue that these forms of data justice are necessary for effective future systems.

**Policy Implications:**

Engaging stigmatised communities—including in relation to gender, sexuality, sex work, drug use, HIV—requires a commitment to data justice. The design and implementation of digital health systems requires investment in ongoing and meaningful consultation with communities and representative organisations.

## Background

In 2018, Australia’s national patient electronic health record system, ‘My Health Record’, attracted considerable public attention following a decision by the federal government to change from an opt-in to opt-out system, with trenchant criticism from privacy and consumer advocates (see Davis et al., 2022; Lupton, 2019). Advocacy organisations representing people living with HIV, people who use drugs and sex workers were critical of these changes, citing concerns about privacy, security and secondary uses of data in relation to HIV status, sex work and drug use (NSW Users and AIDS Association (NUAA), 2018; Positive Life NSW, 2018; Scarlet Alliance, 2018). Following initial criticisms of the My Health Record changes—which included a deadline for all Australians to opt out and ambiguities about how data would be shared—the federal government made amendments which addressed some of the criticisms (My Health Records Amendment (Strengthening Privacy) Bill, 2018). Despite improvements to privacy and control of data, some organisations still held concerns about My Health Record:

Positive Life still takes the position that PLHIV [people living with HIV] from vulnerable populations, such as sex workers, people who inject drugs (PWID), people who live with a criminal record or involved with the criminal justice system, people who are non-monogamous, polyamorous or single, and anyone who receives a STI diagnosis and treatment every 6-12 months could be better off to cancel or delete their MHR [My Health Record]. (Positive Life NSW, 2019)

Although community health organisations like these acknowledged some benefits of My Health Record—such as centralising the storage of health information for coordinated care and providing ease of access to medical information—their concerns about heightened risks for marginalised populations regarding confidentiality, privacy, sharing of data and re-identification of data were seen to potentially outweigh these benefits. In contrast to the position that My Health Record is potentially unsafe for some populations, the Australian Digital Health Agency (2017)—the statutory body who delivers My Health Record—framed ‘digital information [as] the bedrock of high quality health care’ (p. 3), imagining a society in which healthcare is optimised for all by uses of digital information.

### Critical Digital Studies

Digital technologies for supporting or promoting health—including electronic health records, mobile applications, social media sites, wearable and self-tracking devices and virtual health consultations—have become a key part of the landscape of health care and health promotion (Lupton, 2018). Investments in digital health infrastructures by governments and tech industries have been accompanied by optimistic promises of digital health, including the potential for saving healthcare costs, increased efficiency, reducing medical errors and providing greater patient control and transparency (Australian Digital Health Agency, 2017). While these possibilities might eventuate through electronic health records, reviews of the Australian system suggest these benefits are unclear or require further evidence (de Mesquita & Edwards, 2020).

In this article, we take a critical perspective on digital health, which involves challenging ‘technoutopian portrayals’ of digital technologies, and attending to its ‘unintended consequences and potential to entrench existing sociocultural disadvantage or social marginalisation’. (Lupton, 2018, p. 3). The proliferation of digital health has occurred alongside immense growth in health-related population surveillance by state agencies and industries (French & Smith, 2013), in which human life is increasingly quantified and only valued insofar as it can be enumerated, a process described as ‘datafication’ (Mejias & Couldry, 2019; Van Dijck, 2014). Digital health technologies raise potential legal and human rights concerns related to data breaches, privacy, autonomy and ownership of data, and these risks are amplified for marginalised communities (Davis, 2020). However, exclusion from systems and technologies is also a source of potential harm, contributing to ‘data marginalisation’ (Storeng et al., 2021), a longstanding concern in LGBTQ + health advocacy (Davis et al., 2022; Guyan, 2022).

### Digital Health Systems and Marginalised Communities

The impacts of digital health on marginalised communities are important to consider because of their differing experiences of health care. People living with HIV, sex workers, people who inject drugs, gay and bisexual men and transgender and gender diverse people widely experience stigma and discrimination in healthcare settings that can compromise their health outcomes (Broady et al., 2020). These populations may be in regular contact with health services for a range of reasons, including regular sexual health screening, access to gender affirming hormonal therapies, HIV treatment or opiate substitution therapies.

Because of the paucity of social research in Australia documenting the views of these communities regarding digital health systems, the forms of policy and practice that seek to engage them is also underdeveloped. International research has documented concerns about privacy risks of electronic health records in the USA, including amongst trans and gender diverse people (Kronk et al., 2021; Thompson, 2016) and in gay men (Stablein et al., 2015). A US study of men who have sex with men, including a large proportion of Black and Latino men, found that while most were supportive of the potential collective benefits of ‘big data’ and technology to provide important services to underserved populations, they were also concerned about security issues in these systems (Mootz et al., 2020). A study of people living with HIV and physicians conducted in France found both enthusiasm and scepticism for electronic health systems, and the authors categorised different groups of people living with HIV as ‘eHealth believers’, ‘technology skeptics’ and ‘internet adopters’ to characterise these differences (Jacomet et al., 2020). Through a co-design process to develop a prospective mobile health platform for people living with HIV across the EU, Marent et al. (2018) argued that ‘ambivalence’ best characterised the divergent ways people living with HIV felt towards particular features of the platforms (e.g. some trusting and valuing instaneity but not connectivity). They argued that neither ‘resistance’ nor ‘acceptance’ was sufficient to characterise participants’ views of digital health. Advocacy and community responses to growing uses of big data in US HIV surveillance, prevention and care also demonstrate concern about the potential harms for marginalised groups affected by HIV (including trans people, see Minalga et al., 2022), and represent persistent forms of distrust of public health institutions and the harms of lack of engagement with people living with HIV (Bernard et al., 2020; Molldrem et al., 2022). As Michaud et al. (2022) have argued, people who use drugs are subject to a broad range of expanding surveillance practices and datafication throughout harm reduction services and clinical care, some of which are mundane and likely harmless and others of which can exacerbate distrust, proliferate criminalisation and compromise care, especially if shared across systems. Across this literature, there are a variety of themes characterising how marginalised communities engage with digital health systems: tensions between optimism and pessimism about benefits of systems, the importance of transparency, meaningful inclusion and co-design in health surveillance, and distrust of institutions charged with collecting, using and implementing digital systems and keeping data private and secure.

In this article, we focus on key marginalised populations in relation to digital health systems—people living with HIV, sex workers, people who inject drugs, gay and bisexual men and transgender and gender diverse people. Although distinct groups, we focus on these groups collectively for two key reasons. Firstly, in Australia, these groups are listed together as priority populations in national strategies aimed at addressing the prevention, treatment, care and stigma of bloodborne viruses (BBVs) and sexually transmissible infections (STIs), which coordinate the allocation of funding for health services and other support (Australian Department of Health, 2018). For example, priority populations may be in regular contact with a similar range of health services, including for sexual health screening, access to gender affirming hormonal therapies, HIV treatment or opiate substitution therapies, and the professionals working with and advocating for these populations are supported and connected through the same funding streams and shared domains of expertise. Secondly, and as indicated above, advocacy groups representing some of these priority populations raised a similar set of concerns about My Health Record when it was changed to an opt-out system (NUAA, 2018; Positive Life NSW, 2019; Scarlet Alliance, 2018). Overall, the priority populations we discuss are impacted by interrelated experiences of stigma and discrimination in relation to practices and subjectivities perceived as socially ‘deviant’ (Callander et al., 2019; Couto e Cruz et al., 2018; Northrop, 2017; Perales, 2018; Platt et al., 2018; Reisner et al., 2016). Although interrelated, the extent to which these forms of stigma are experienced differs markedly in Australia, with sex workers and people who inject drugs reporting more frequent experiences of discrimination in healthcare, compared with gay men and people living with HIV (Broady et al., 2020). The extent to which people experience marginalisation can also be exacerbated by multiple and cumulative structural factors, including racialisation, poverty, migrant status and criminalisation (Bernard et al., 2022; Couto e Cruz et al., 2018; Newman et al., 2021; Selvey et al., 2018; Stangl et al., 2019).

Australia’s national electronic health record system promises public and individual health benefits, but its utility and safety for groups who are often stigmatised in healthcare settings is questionable and possibly counterproductive (Cruz, 2022). It is vital also that the development of digital health is guided by consultation with these particular communities and their advocates. This paper focuses on how key informants—holding expertise in digital health and the practical, ethical and political complexities of engaging communities vulnerable to stigma and discrimination—conceptualised engagement with digital health systems and how potential benefits and safety for stigmatised communities might be optimised.

## Methods

### Design

The material presented here was collected as part of the ‘Trust in Digital Health’ study, a pilot study funded by the Australian Government Department of Health. The project documented community and expert views on trust and engagement in digital health for communities affected by BBVs and STIs, as identified in national strategies (Australian Department of Health, 2018). The study methods comprised semi-structured qualitative interviews with key informants and a national online cross-sectional community survey. The study design and materials as well as analysis outcomes were developed in partnership with community organisations representing communities affected by BBVs and STIs (see acknowledgements). Selected findings have been made publicly available (Newman et al., 2020). This paper reports on the key informant interviews.

### Participant Recruitment

Individual, semi-structured interviews were conducted with purposively selected key informants, including professionals working in advocacy, policy, research, health promotion and/or clinical practice. Participants were identified through formal and informal networks and were eligible to take part if they agreed that they held professional knowledge relating to either or both digital health and policy and practice relating to communities affected by BBVs and STIs, and were aged 18 or over, a resident in Australia and proficient in English. Potential participants were invited to participate via email. No compensation or other incentives were provided as part of the study. Of the 23 prospective participants who were invited, 16 agreed to participate.

### Human Subjects Protection

All participants provided audio-recorded consent prior to taking part in interviews. The study protocol and procedures were approved by the UNSW Human Research Ethics Committee (HC191000) and the ACON Research Ethics Review Committee (RERC 2019/29).

### Data Collection

The interview guide was developed specifically for the study, in consultation with the research team, which included representatives of partner organisations. The guide explored participant understandings of trust and digital health, beliefs about how digital health systems are viewed by communities affected by BBVs and STIs, perceived benefits and risks of these systems for communities and potential solutions to optimise and improve engagement with the systems. Interviews were conducted by CN between March and June 2020 by telephone or Zoom. Participants were asked to provide key demographic details, summarised below. Interviews lasted an average of 48 min (between 20 and 67). Transcripts were de-identified, removing names, places and organisational roles. Data collection for this study occurred during the early stages of COVID-19 restrictions in Australia (beginning in mid-March 2020), which impacted on the capacity of some people to participate.

## Participant Demographics

The demographic characteristics of the 16 participants are shown in Table 1. Participants worked primarily in peer-based non-government organisations, across advocacy, education and policy, and holding expertise in the experiences and needs of trans and gender diverse people, people living with HIV, sex workers, gay and bisexual men and people who inject drugs. The participants were diverse in gender, and a relatively high proportion of participants identified as queer, gay or bisexual/pansexual. Almost all participants identified as White or Anglo, and held higher education qualifications. To protect confidentiality in a small sector of professionals, participants are only referred to in this paper by their interview number, e.g. [P01].

**Table 1:** Participant professional and demographic characteristics

*Participant characteristics*	*N* = 16
**Decade of birth**	1960s (*n* = 3), 1970s (*n* = 4), 1980s (*n* = 9)
**Time working in the area**	Range of 6–32 years (average mean of 16 years)
**Organisation type** (not mutually exclusive)	Peer-based non-government organisation (*n* = 10), University (*n* = 4), consultant (*n* = 2), health service (*n* = 1)
**Expertise with marginalised communities** (not mutually exclusive)	Trans and gender diverse people (*n* = 9), people with HIV (*n* = 8), sex workers (*n* = 7), gay and bisexual men (*n* = 7), people who inject drugs (*n* = 6)
**Professional role** (not mutually exclusive)	Advocacy (*n* = 10), Education (*n* = 7), Policy (*n* = 7), Research (*n* = 6), Health promotion (*n* = 3)
**Gender**	Cis woman or female (*n* = 7), cis man or male (*n* = 6), non-binary (*n* = 2), trans man (*n* = 1)
**Sexuality**	Queer (*n* = 5), gay (*n* = 4), bisexual/pansexual (*n* = 4), straight (*n* = 3)
**Cultural/language background**	Anglo/Irish/European (*n* = 13), Asian or Pacific Islander (*n* = 2), Aboriginal or Torres Strait Islander (*n* = 1)
**Highest qualification**	PhD (*n* = 4), Masters (*n* = 6), Graduate Diploma (*n* = 2), Bachelor (*n* = 1), Diploma (*n* = 1), High School (*n* = 2)

### Data Analysis

The author team are experienced working with and researching aspects related to communities affected by BBVs and STIs, and most of the authors identify as members of one or more communities related to the study. Consequently, the assumptions and priorities we brought to the analysis were explicitly shaped by an investment in valuing and seeking to improve the lives of these communities. Transcripts were analysed using reflexive thematic analysis (Braun & Clarke, 2019), in which themes were developed through a combination of our theoretical approaches, research questions, understanding of literature and the interview material. A coding framework was developed from the study design and refined with new codes through several rounds of inductive coding. AS led the process of coding, and discussed coding amongst authors to ensure sufficient variation and depth was achieved, including through an analysis workshop with the full team of researchers and community partners. Data were manually organised into codes using QSR NVivo (12.6.0). As the themes were iteratively developed, and the manuscript drafted, interview transcripts were read again to ensure that the analysis reflected participants’ accounts. Although participants spoke about a broad range of digital health systems, electronic health records occupied a central focus amongst participants. This paper reports on three key themes that explore how electronic health records could be made more useful and safer for communities affected by BBVs and STIs.

## Findings

### An Unmet Promise: the Potential Benefits of Electronic Health Records

Participants cited numerous potential benefits of electronic health records but these were typically seen as speculative and anticipatory, hinging on the *promise* of digital health innovations (Lupton, 2018). P12 explained: ‘So there are lots of promises [of digital health], but the promises come with all sorts of caveats and […] the reality hasn’t matched the promise’. Many participants wanted electronic health records to be beneficial and practical for the communities with whom they worked. However, they described the roll-out of My Health Record and the decision to enrol all Australians in the system unless they opted out as a policy failure which had neglected consultation with key communities affected by stigma and discrimination in healthcare systems:

the way that the government looked to implement that without consultation with our communities, and it was done in a really back-handed way, we ended up having to do something we didn’t want to do, which was actually advise our community to withdraw from it. And that was very conflicting ‘cause actually I think that electronic health records, in an ideal, perfect world, where there was no stigma and so on, would be perfect for our community [of people who inject drugs]. [P09]

Despite taking a critical perspective, this participant imagined an electronic health record system as potentially utopian, as long as it was able to be implemented in a social world without stigma. Similarly, P15 explained that, ‘ignoring the […] potential for discrimination, there’s a benefit for the trans community, in terms of access to and having information stored through My Health Records around gender-affirming care. ‘Cause we know people move house, have interruptions to their care’. Imagining potential technological benefits required participants to deliberately suspend their disbelief about the context in which systems were designed and delivered, and to instead imagine a world in which communities did not face discrimination and stigma.

Participants also spoke about the potential of electronic health records to provide health benefits not only for communities affected by BBVs and STIs, but for the whole population. As P01 summarised it: ‘[A] single, electronic, health record is the Holy Grail: continuity of care, avoid repeated tests, avoid wrong prescriptions, etc.’ Participants emphasised that efficiency, cost-saving and convenience were key benefits that a universal electronic health record could theoretically offer to everyone (ADHA, 2017). This implied support for the technoutopian potential of an electronic health record system, but also recognition that existing systems for managing patient information were fragmented, unreliable and frustrating (Lupton, 2018). In this context, an electronic health record was felt to be particularly important for assisting individuals in their communities who managed multiple and complex healthcare conditions. Given that many participants were also members of the communities for whom they advocated, some provided reflections on their own experiences with (digital) healthcare to contextualise their advocacy. For example, P03 reflected:

… our health system is so fragmented and my memory is so bad, and there are days when my experience of mental illness means I have trouble telling a linear narrative, and sometimes even trouble speaking. And I see different doctors for my general health, for my mental health, for my sexual health. I now have to see a tertiary specialist for something else and the person who manages or oversees my continuity of care is me. So, in theory, a personal, person-controlled, electronic health record should just let me say, “Yes, I saw a doctor on this day. This is the diagnosis that I got. This is the medication that I’m on,” and that that would go into a kind of broad summary that I can provide when I see my tertiary specialists once every 12 months.

While this participant could imagine personal benefits in an electronic health record system, they did not believe this had been achieved with the Australian system.

Beyond speculating about personal and direct benefits of electronic health records, some participants imagined that data had the potential to be useful for surveillance and research and, therefore, valuable for the development of tailored service provision and advocacy activities for communities affected by stigma in healthcare. P06 explained that in Australia, ‘we don’t currently have meaningful or accurate population estimates on LGBTI people’, and argued that digital health systems which stored information on sexual orientation, gender identity and sex characteristics could potentially provide useful understandings of population size and need. P03 imagined a system that could identify gaps in: ‘different conditions or different demographics with unmet needs. And on that basis, from that you get things like funding, recognition, inclusion in research’. The promise of digital health systems therefore extended from increasing service efficiencies to achieving the much broader social good of improved visibility and inclusion for communities who have been largely overlooked in standardised data collection systems (Cruz, 2022; Davis et al., 2022; Guyan, 2022). These imagined benefits of increased dataveillance contrasted with risks related to datafication and privacy, as we explore in the next section.

### A Leaky System: the Ever-Expanding Risks of Electronic Health Records

While participants viewed the potential benefits of electronic health records as unrealised in practice, the risks for communities affected by BBVs and STIs were highly salient to participants, even if these risks were also speculative. In contrast to imagining digital health futures, the discussion of harms were frequently based on examples of discrimination or breaches of privacy experienced in healthcare settings.

A common narrative was ‘trans broken arm syndrome’, which referred to the experience amongst transgender people of seeking health care for something not related to gender—such as a broken arm—and finding that their health care providers had become inappropriately preoccupied by their gender history. P10 explained:

If you do pursue a physical transition, you can still have health records that are in your old gender, that are floating around. It becomes really problematic if, for example, you’re admitted into emergency and you’re unconscious, and somebody pulls up your Medicare [public health insurance record] for whatever reason. And then everybody gets really confused and starts focusing on [your gender] as opposed to anything else.

Other participants raised a similar phenomenon in relation to patient histories of injecting drug use, STI testing or diagnoses with HIV or hepatitis C. Participants believed that it was important for individuals to have agency over the disclosure of such sensitive information in health care consultations so that they could avoid or mitigate the potential for discrimination or refusal of service.

While the ideal of integrated health services is posed as a key potential benefit of a national electronic health record (ADHA, 2017), it ignores the fact that not all citizens receive safe, non-judgemental care. Participants noted that some communities commonly experience judgement and prejudice in healthcare settings (Broady et al., 2020), which undermined trust in biomedical expertise and healthcare:

I don’t think there’s any such thing as full trust between patients and doctors. There are functional levels of trust. But that usually is only one-to-one and I think the fear is when you put your trust in someone and they pass the records on or pass information on about you cunningly or wittingly to someone else. […] Do we owe our healthcare providers 100 per cent transparent insight into us, our life system? Do we really owe them that for them to do their job? I might be a dedicated heroin and meth user for 20 years and 10 years later not have done it for 10 years. There’s so many reasons that is not relevant to going to a doctor. […] I cannot think of any circumstance in my life [or] the lives of anyone I know that I’ve talked to about this, where being 100 per cent honest about my drug use, my current drug use, where [that] has been a positive thing. [P09].

P09 challenged the assumption that comprehensive medical and lifestyle information is necessary for achieving safe and quality care, especially when types of data (e.g. about stigmatised practices or identities) can become unsafe if shared with other clinicians or services. Participants also argued that expecting people to trust health systems is not realistic when individuals have become wary due to the experience of discrimination and stigma. Similarly, participants believed that their communities would find it difficult to trust governments to act as custodians of highly sensitive data. Hacking and data breaches in government data systems were nominated as evidence that governments could do little to protect data, for example ‘the Singaporean case where a whole bunch of information about people living with HIV was leaked’ [P02]. Participants also raised concerns about whether data in electronic health records could be linked to other systems which patients did not know about, such as private health insurance companies, social services, law enforcement agencies, and employers.

Participants explained that criminalisation related to drug use, and laws related to sex work and HIV disclosure (which vary across Australia) played a significant role in making digital health systems potentially unsafe for some people. P16 suggested that the police and legal threats were amplified for people whose lives were intersected by multiple health and social inequities, ‘Especially if you have intersections like HIV status and sex work. And, you know, the crime of being Black, Indigenous’. Other participants argued that those whose livelihoods and citizenship rights were precarious, such as migrant sex workers, faced severe criminalisation impacts. Participants therefore believed that if healthcare systems stored data on criminalised practices, communities would be less willing to engage healthcare, even if data was deidentified. P14 explained that a criminal record, ‘no matter how old it is, hangs around and follows you’. There was belief that institutions could not be trusted to protect the individual:

If you’re disclosing something that relates to criminal or otherwise stigmatised activity and you have children, people are genuinely worried about that being reported to child services. And maybe being reported to law enforcement. It happens now because health professionals do make such referrals. So I guess it’s just the unknown of who else may have access to this information and they [may] exercise their discretion to take action against me. [P07]

Because fears of criminalisation intersected with stigma and discrimination, some participants were particularly troubled by increased dataveillance. Regarding Australian approaches to surveillance, P13 explained.

we have an extremely long history, since the mid-nineteenth century, of directed use of [health surveillance] data to control, exploit or exterminate populations that are determined by whoever the state actors are of the time or private interest actors to be ‘undesirable’ in a whole variety of physical and social ways.

Based on these and similar perspectives and coupled with weak data protection policy and legal frameworks, participants discussed how data could be used as a means of coercive control. Some participants were concerned that the collection of sensitive data in systems like My Health Record could be misused, if not now, in the future. In contrast to the first theme, these participants expressed fears of a dystopian future of amplified discrimination and social control of stigmatised populations.

Due to the impacts of ongoing criminalisation and persistent stigma and discrimination, participants indicated that digital health systems were not likely to be embraced by the communities with which they worked. However, as we explore next, participants proposed suggestions to help increase the utility and safety of these systems.

### Consent and Consultation as Solutions for Imperfect Digital Health Systems

Participants identified numerous strategies for reforming digital health systems to secure benefits and promote the security, privacy and dignity of their communities, including exploring consent and data ownership principles and practices (Molldrem & Smith, 2020; Taylor, 2017). Participants commonly framed consent as a specific, informed and dynamic practice for individuals asked to provide data. For example, P04 asked ‘what am I consenting to when I sign up? And is the consent explicit and ongoing?’ Similarly, P02 reflected:

there’s not a lot of discussion around data sovereignty and who owns that data, who’s responsible for that data. And, if I’m going to give you my data, what controls do I then have over it? Can I pull out at any point in time?

Participants, however, were also cautious about simplistic notions of individual consent. They recognised that individualised consent could intensify the assumption that data privacy and security was a personal responsibility, obscuring, therefore, the interlinked data governance and sharing structures of big data systems. Engaging in an informed manner with these complexities for disclosure and consent would require a high degree of health literacy and significant labour on the part of the patient, some of whom may be preoccupied with severe illness. In this view, consent could shift the burden of data safety onto an already burdened individual. As P12 put it, focusing debates about consent, data security and privacy at an individual level perpetuated a ‘fantasy of the well-informed health consumer who is able to navigate that system and make all the appropriate judgements’. Participants argued that to overcome these pitfalls, the development of much more effective methods for aligning consent, disclosure and data safety were needed in digital health. For example, P03 was critical of the use of participant information sheets for securing consent for research and lengthy terms and conditions agreements in which ‘the initial impulse is to give people the 25-page explanation of all of the ways in which data is going to be used so that people are informed; that very kind of enlightenment, contractual model’. Instead, P03 argued that consent needed to be directly linked with healthcare, particularly as it was difficult to consent in the abstract without having first experienced how a data collection system works. They explained: ‘I can’t really consent until I’ve actually seen the system that I’m trying to use. And, if I don’t get the feeling that it’s trustworthy, then I will figure out how I can delete my account’. Participants held mixed views about the types of consent mechanisms that would work in practice and recognised that consent was not a panacea. They nonetheless wanted to see expanded and dynamic processes of consent explored and debated.

Strategies for strengthening consent were not only technical, they also implied radical transformation of the values underpinning system design and required healthcare institutions to acknowledge the ‘deep distrust between communities and the medical system’ [P09] and other powerful institutions, particularly governments. Consequently, participants wanted to see meaningful co-design of digital health systems, including consumer and community engagement methods to create systems that were valued and supported by communities affected by BBVs and STIs. P03 felt that this type of consultation rarely happened: ‘nobody really ever seems to ground [digital health] in actual consultation with communities or research into what people think’. Participants emphasised that engagement needed to occur at all steps of the conceptualisation, design and delivery and monitoring of systems. Some were particularly critical of government-led consultation attempts that had been ‘tokenistic’ and in which ‘consumers are heavily engaged, they’re given really strong feedback, and then things have gone in a complete opposite direction despite all the feedback from consumers’ [P02]. Similarly, P04 reflected on the My Health Record system when it transitioned from opt-in to opt-out: ‘and we’ve got this many weeks to respond before there’s a cut-off, before everyone gets a record created. And it’s like, well, how do you co-design in that?’ Participants suggested that authentic consultation, including the allocation of sufficient time and money to community-based organisations, could allow communities to contribute to solutions that promoted safer, trustworthy systems.

## Discussion

This paper analysed key informants’ views regarding benefits and harms of digital health systems for stigmatised communities, and opportunities for creating conditions to build trust, utility and safety for digital healthcare systems. Participants were optimistic that digital health systems could improve the health and rights of communities affected by BBVs and STIs. However, these benefits were largely promissory and rarely manifested in the lived experience of affected communities. Moreover, appreciating what digital healthcare might achieve required an imaginary society that was free of discrimination towards stigmatised communities and criminalisation of certain practices. Critical digital health scholars have noted that data technologies are commonly presented as promising technoutopias and on that basis are justified for the future economic and social benefits that they might yield (Lupton, 2018). However, technoutopian portrayals of digital health erase the realities of stigmatised communities, and are not likely to be useful and may instead be harmful.

Participants offered nuanced and practical concerns and suggestions regarding data privacy and security. They cautioned against oversurveillance (although some could also see opportunities for understanding their communities with better dataveillance) and described instances in which risks had eventuated for individuals in the communities they served. Consequently, participants noted that members of their various communities were often reflexively wary and unlikely to wholeheartedly trust digital health systems. It is well-documented that marginalised populations undertake significant work to manage disclosure of identity and practices in healthcare settings, segment care between different providers, or do not engage in healthcare altogether to avoid discrimination and maintain privacy (Broady et al., 2022; Davis & Manderson, 2014; MacKinnon et al., 2020; Northrop, 2017). These concerns need to be addressed for digital health to deliver its promises for communities who regularly experience stigma in healthcare settings. However, participants suggested that positive steps could include investment in consent, protection of privacy, clarity about data uses and commitment from government to invest in authentic consultation with marginalised communities in order to better design and reform digital health systems.

Key informants indicated that the criminalisation of practices related to drug use and sex work occupied a key barrier to trust and engagement with digital health systems for some people, especially if needing to discuss these practices when accessing healthcare. Criminalisation of practices related to drug use and sex work vary across Australian jurisdictions. For example, each state in Australia employs a combination of criminalisation, licensing and decriminalisation models for sex work, which also vary within a jurisdiction depending upon other factors, including street sex work and living with HIV (Scarlet Alliance, 2014, p. 6). Similarly, laws regulating the personal use and possession of illicit drugs vary across Australia (Hughes et al., 2018), although peer distribution of injecting equipment is universally prohibited outside of pilot programs (Lancaster et al., 2015). Key informants recognised that some people were more vulnerable to criminalisation because of intersecting identities, for example migrant sex workers and sex workers living with HIV (Hoppe et al., 2022; Selvey et al., 2018). Key informants indicated that greater sensitivity and security were needed for the inclusion of data indicating practices that are criminalised in digital health systems (Michaud et al., 2022; Molldrem et al., 2022). They also indicated that decriminalisation of these practices could improve engagement and trust in digital health systems, but that the issue of discrimination and stigma in healthcare settings may persist.

Electronic health records which contain information about identities and practices (e.g. trans identity or injecting drug use) that attract social proscription were perceived as a threat to healthcare and wellbeing and, in some circumstances, income and citizenship status. A common view was that governments were unable and/or unwilling to protect the interests of all citizens expected to use data systems. These harms counteract what appears to be the utilitarian assumption that figures in the roll out of digital health data systems, that is, the ends justify the means. Our analysis indicates that digital health may not, on balance, deliver benefits in an inclusive and comprehensive manner. In addition to the key informants’ perspectives, through our national online cross-sectional community survey of the general population and priority populations undertaken in early 2020, we found that sex workers, trans people and people living with HIV were more likely to report opting out of My Health Record than the general population and gay and bisexual men (Newman et al., 2020). Common reasons for opting out included concerns about being treated poorly in healthcare settings, concerns about privacy and security and sharing of data for commercial purposes or with other government agencies, and these concerns were heightened for sex workers. While it is not clear whether these anticipated risks (from both the survey and findings explored in this paper) have played out in relation to My Health Record, current data suggests that the system is not well-utilised by patients and that clinicians have mixed views about its value in delivering patient care (ADHA, 2022; Davey, 2022; Mullins et al., 2021). Although My Health Record was a particular focus for participants—because it was a recent controversy in the Australian digital health landscape—our findings provide insight into the broad issues of engaging communities affected by BBVs and STIs with digital health systems.

Beyond the specifics of My Health Record, the pitfalls and common narratives noted by participants—for example, the ‘trans broken arm’ narrative—offer many points of entry for considering how digital health care could be reimagined as safer and relevant to communities. We construe these reimaginings of digital health as data justice, which involves applying a social justice lens to studying issues of datafication and challenging the institutions, practices and processes through which data shapes society and modes of belonging and citizenship (Dencik et al., 2022). Beyond technoutopian portrayals which constitute new ways of generating and using data as opportunities for improved economic and human development (Lupton, 2018), data justice is concerned with how data and digital systems relate to basic freedoms and human rights (Taylor, 2017). The rapidly increasing datafication of social worlds call for forms of data governance that avoid and mitigate loss of privacy and the inequities that might arise through the commercialisation of personal data (Davis, 2020). Simultaneously, data justice involves conceptualising how communities can resist and reformulate data practices to collective public benefit and afford people greater control over how their data is used in healthcare, or to enact informed refusal of systems (Benjamin, 2016; Molldrem & Smith, 2020). In this study, participants were engaged with these matters of data justice in relation to the communities they worked with and represented. They indicated that inclusive digital healthcare policy settings and practices are achievable and necessary for creating the conditions under which diverse communities stigmatised by BBVs and STIs can meaningfully and safely engage with either existing or emerging approaches to capturing, storing and sharing personal health data. However, beyond simply ‘fixing’ these systems, participants indicated that the material and structural issues affecting their communities must also be considered and addressed to fully realise health equity (Cruz, 2022).

In addition to addressing legal and other structural barriers impacting the communities we focused on in this article—including criminalisation of drug use and sex work, and the stigmatisation of ‘deviant’ subjects in healthcare—exploring consent mechanisms and committing to community consultation were raised as necessary to better engage communities with digital health systems. Key informants framed consent as needing to be informed, dynamic and specific, with the possibility of opting out and refusing (Benjamin, 2016; Budin-Ljøsne et al., 2017). However, some participants also observed that consent processes (including both consent in research and ‘terms and conditions’) can be highly technical, contractual and individualistic, and that meaningful consent might be better conceptualised as relational, involving trust, reciprocity and obligations between communities and institutions who are stewards of data (Dixon-Woods et al., 2017; Kadam, 2017; West-McGruer, 2020). Although My Health Record affords some dynamic consent mechanisms, these typically rely on a digitally literate and engaged consumer (Davis et al., 2022). These tensions regarding consent sit uneasily with a national system that is unlikely to incorporate individual needs, but the problems nonetheless underscore the importance of trust in the context of (digital) health systems (Adjekum et al., 2018; Calnan & Rowe, 2008).

Key informants argued that meaningful involvement of communities in the design and delivery of digital health systems was necessary to build trust and to ensure that the diverse needs of communities could be incorporated into systems, ideally resulting in safer, and more relevant systems (Marent et al., 2018). These elements of consultation and engagement reflect the principle of meaningful involvement of communities affected by HIV, originating from the advocacy of people living with HIV since the 1980s (Spieldenner et al., 2022). Our findings suggest that community members and experts working in advocacy and community-based organisations should be meaningfully consulted with and resourced to support the design and implementation of functional, equitable and safe digital health systems. Given that community-based and not-for-profit sector organisations are involved in generating data about the populations they engage (Michaud et al., 2022), there is also scope to build and resource data capabilities within organisations (McCosker et al., 2022). While meaningful engagement and resourcing are important, we should also be wary of technical solutionism to structural issues (including social stigma) and support a continued care regarding the open question of whether digital technologies are always necessary or beneficial to communities (Dencik et al., 2022; Lupton, 2018).

A clear limitation of this study is that we only interviewed key informants. These participants held decades of expertise working with their communities, and many participants also belonged to one or more of the communities they worked with. However, participants sharing belonging may be more privileged in comparison to some community members they represent because of their professional role, educational attainment, country of birth and cultural or racial background. In particular, we note that our sample was not particularly culturally diverse, as participants were primarily Anglo-Australian. Another limitation is that our sample may be viewed as overly cynical about digital health systems. However, the participants we interviewed were neither disengaged with digital health systems nor critical for the sake of it—they were aware of what was lost by systems not being collaboratively designed and implemented, and wanted future systems that could help improve the health of their communities.

## Conclusion

Our findings suggest that digital health system design and implementation should be conducted in ways that are sensitive to the risks that particular communities can face in the expansion of digitally integrated healthcare systems, including privacy, consent, discrimination, stigma and criminalisation. Resources should be directed towards remediating the social, legal and policy conditions that continue to make it unsafe for some communities participate in and benefit from digital health, and in supporting meaningful consultation with organisations who represent and work with these communities (Cruz, 2022; Davis, 2020; Guyan, 2022). This entails prioritising principles of data justice in digital health (Dencik et al., 2022; Molldrem & Smith, 2020; Taylor, 2017), including moving beyond technoutopian portrayals of digitisation in public health (Lupton, 2018). Policymakers and other actors should prioritise data justice and meaningful consultation in all efforts to design and operationalise digital health systems. Further research should consider how the unintended consequences of digital health systems impact on and disadvantage other communities.

## Data Availability

Data are not shared.
